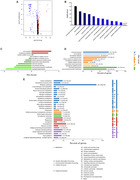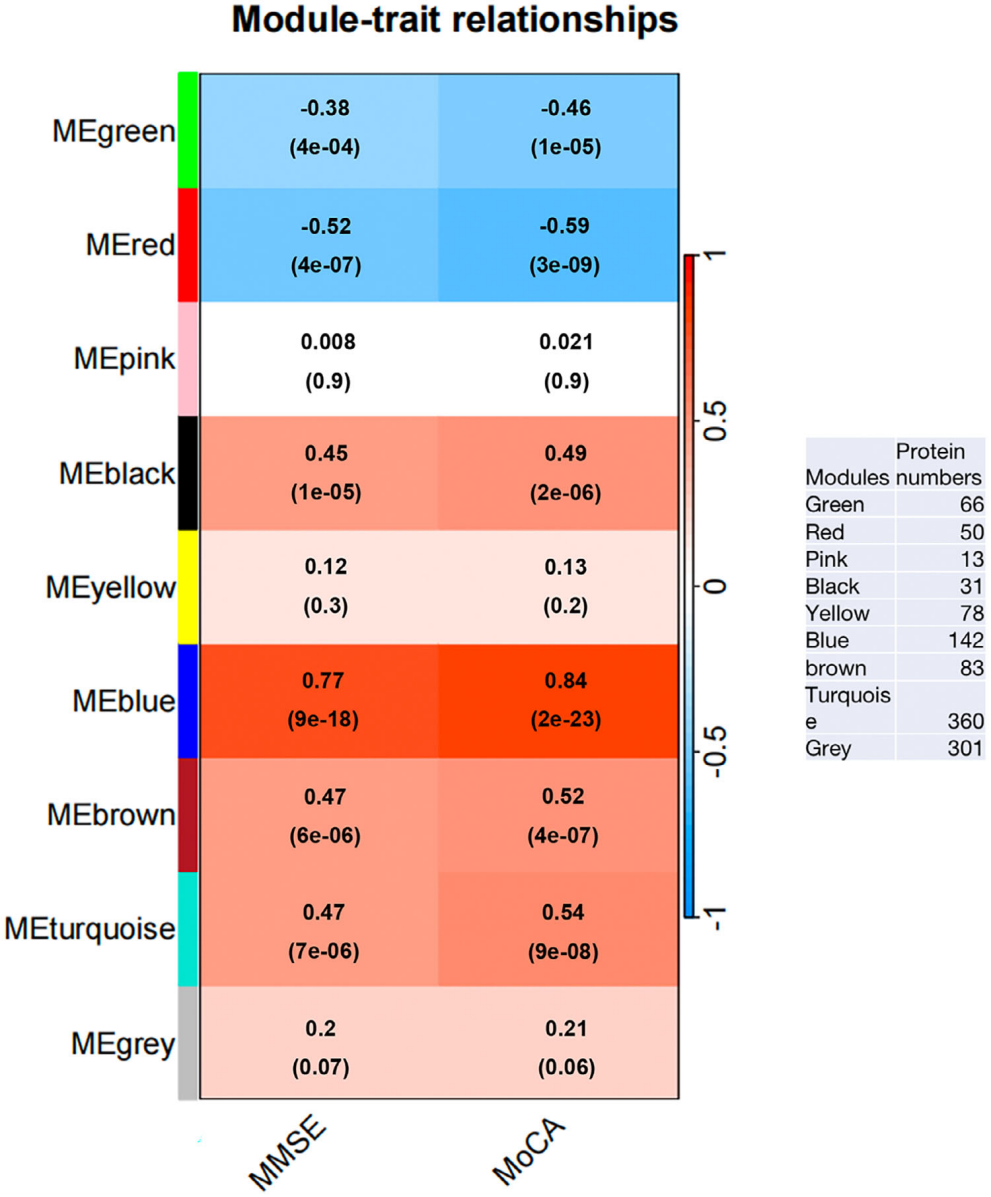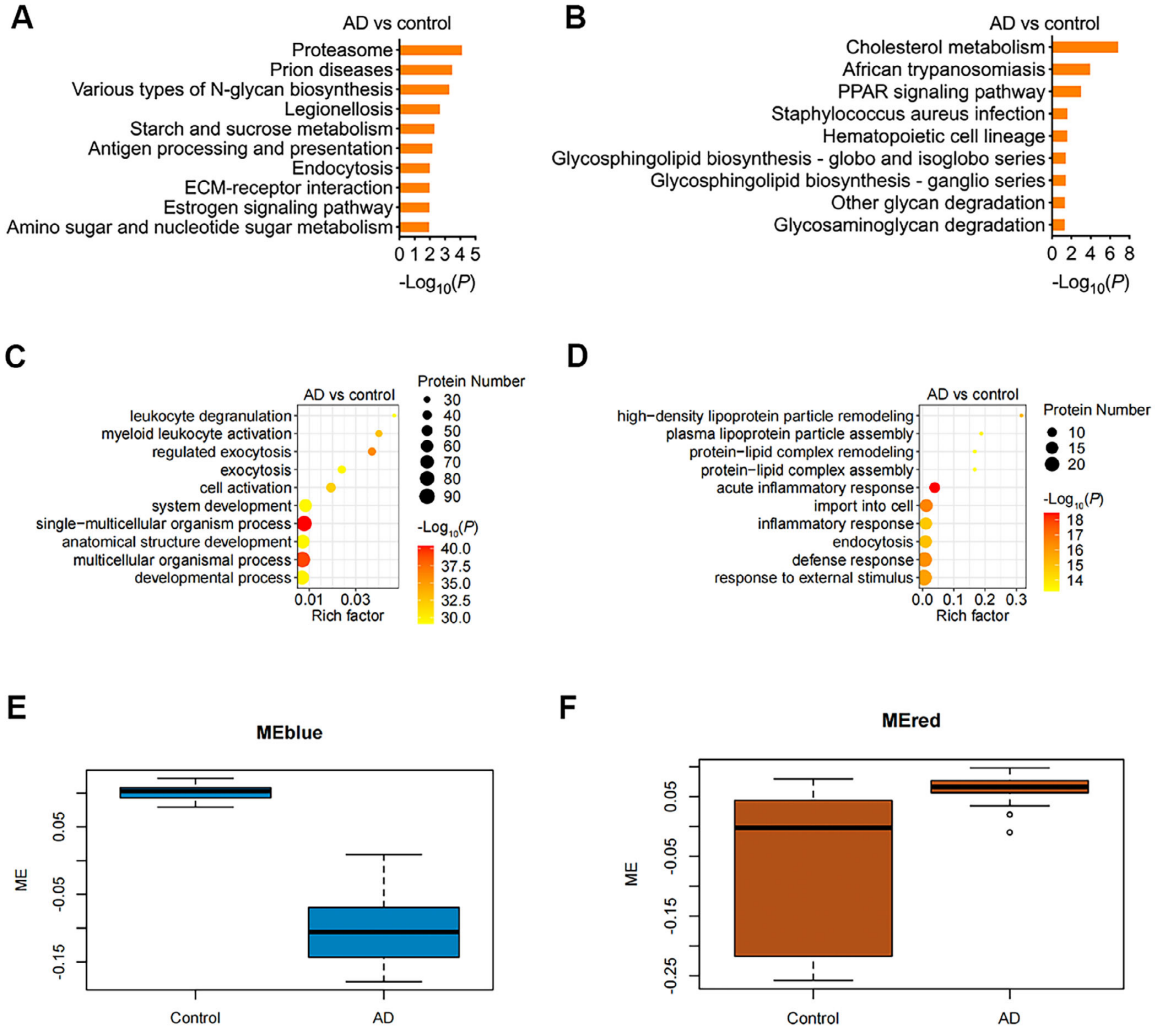# High‐performance plasma proteomic biomarker candidates in early Alzheimer's disease

**DOI:** 10.1002/alz.092774

**Published:** 2025-01-09

**Authors:** Yuhan Xie, Hui Sun, Fang Liu, Yaonan Zheng, Mang Zhang, Xin Ma, Xin Yu, nan zhang, chaolan huang, Huali Wang

**Affiliations:** ^1^ Peking University Institute of Mental Health (Sixth Hospital), Beijing China; ^2^ Clinical Research Institute, State Key Laboratory of Complex Severe and Rare Diseases, Peking Union Medical College Hospital, Chinese Academy of Medical Science & Peking Union Medical College, Beijing China; ^3^ Department of Neurology, Tianjin Medical University General Hospital, Jin Tian China

## Abstract

**Background:**

The present study aimed to establish a panel of plasma proteins that are associated with cognitive performance and amyloid‐β burden and establish their utility for distinguishing early Alzheimer’s disease (AD) from healthy controls.

**Method:**

Plasma from fifty patients with early AD (all amyloid‐positive) and forty‐nine healthy controls (HCs) were assayed using quantitative mass spectrometry. The weighted correlation network analysis (WGCNA) was used to explore the association between plasma protein levels and cognitive performance and filter out the high‐performance plasma proteomic biomarker candidates. Further correlation analysis was used to examine these proteins’ association with amyloid‐β burden in AD brain measured with PET imaging.

**Result:**

We identified 1124 proteins that were dysregulated in AD plasma. Further, 22 panel proteins representative of the AD plasma protein profile were selected based on their association with the cognitive performance and cerebral regional SUVR of PiB‐PET imaging. The panel proteins, involved in the amyloid‐β protein production, metabolism and immune processes, have demonstrated high performance in distinguishing between early AD and HCs.

**Conclusion:**

This study comprehensively profiled the AD plasma proteome and identified 22 dysregulated proteins as high‐performance biomarker candidates for early Alzheimer’s disease.